# Coronavirus research topics, tracking twenty years of research

**DOI:** 10.1038/s41597-025-04992-z

**Published:** 2025-06-10

**Authors:** Amir Aryani, Jingbo Wang, Luis Salvador-Carulla, Jihoon Woo, Cathy P. W. Cheung, Zhuochen Wu, Hui Yin, Junhua Xiao, Elisabeth A. Lambert, Jason Howitt, Jean M. Davidson, Serene Yoong, John B. Dixon, Rachel E. Climie, Jose A. Salinas-Perez, Nasser Bagheri, Celine Santiago, Joanne Williams, Nilmini Wickramasinghe, Leo Ng, Clara C. Zwack, Gavin W. Lambert

**Affiliations:** 1 Swinburne University of Technology, Melbourne, Australia; 2 National Computational Infrastructure, The Australian National University, Canberra, Australia; 3 University of Canberra, Canberra, Australia; 4 Australian National University (ANU), Canberra, Australia; 5 School of Allied Health, La Trobe University, Melbourne, Australia; 6 California Polytechnic State University, San Luis Obispo, US; 7 School of Health and Social Development, Deakin University, Burwood, Australia; 8 University of Tasmania, Hobart, Australia; 9 Universidad Loyola Andalucía, Andalucía, Spain; 10 Victor Chang Cardiac Research Institute, Darlinghurst, Australia

**Keywords:** Medical research, Research data

## Abstract

Research publications aimed at understanding the various aspects of Coronaviruses, particularly COVID-19, have significantly shaped our knowledge base. While the urgency to monitor COVID-19 in real-time has decreased, the continual influx of new research of monthly articles underscores the importance of systematic review and analysis to deepen our understanding of the pandemic’s broad impact. To explore research trends and innovations in this space, we developed a pipeline using natural language processing techniques. This pipeline systematically catalogues and synthesises the vast array of research articles, leading to the creation of a dataset with more than eight hundred thousand articles from July 2002 to May 2024. This paper describes the content of this dataset and provides the necessary information to make this dataset accessible and reusable for future research. Our approach aggregates and organises global research related to Coronaviruses into thematic clusters such as vaccine development, public health strategies, infection mechanisms, mental health issues, and economic consequences. Also, we have leveraged the contribution of health experts to review and revise the dataset.

## Background

The rapid escalation in morbidity associated with the severe acute respiratory syndrome of coronavirus 2 virus (SARS-CoV-2) and the global pandemic led to rapid research on SARS-CoV-2 to help develop health programs to curb the impact of coronavirus disease. The subsequent and ongoing research efforts generated an exponential related literature. The volume and breadth of publications created an imperative to develop data science infrastructure and approaches for rapidly collating, reviewing, cataloging, and disseminating information to varied stakeholders (Table 1). The World Health Organisation^1^ (WHO) and Centres for Disease Control and Prevention^2^ (CDC) developed searchable literature repositories. The Dimensions project produced a dataset comprising published articles, preprints, clinical trials, grants, and research datasets related to COVID-19. The COVID-19 Open Research Dataset (CORD-19) included scientific papers on coronavirus research. It was designed to facilitate the development of text mining and natural language processing research over its collection of metadata and structured papers^3^. While efficiently accessing, collating, and interpreting data from these repositories requires some technical and domain expertise, platforms such as LitCOVID^4^ (LitCovid - NCBI - NLM - NIH) and CoronaCentral^5^ (Dashboard | CoronaCentral) provide accessible dashboards where literature is collated and categorized by type, topic, and author location. At the time of writing, more than 450,000 publications have been presented in these readily accessible dashboards. While articles have been classified and presented as covering a range of research activities, including treatment, prevention, epidemiology, and policy, or according to the field of research codes, the challenge remains to monitor the breadth of literature timely with ongoing spikes in infection and emerging variants^6^ and literature noting health complications, such as long COVID^7^, autoimmune disease development^8^ and cardiovascular complications^9^. Identifying trends accurately and efficiently and retrieving and collating information to inform the community and researchers is crucial. This effort is essential to provide an evidence base to guide public policy and allocate resources effectively.

**Table 1:** COVID-19 literature repositories.

Repository	Coronavirus		Data source		Articles	Status	Updates
	SARS-CoV-2	Other	PubMed	Other			
CORD-19^3^	*✓*	*✓*	*✓*	*✓*	59,887	Achieved	Final version: July, 2022
WHO COVID-19^1^	*✓*		*✓*	*✓*	878,426	Achieved	Weekly (until June 2023)
CDC COVID-19^2^	*✓*		*✓*	*✓*	1,933	Achieved	Weekly (until July 2023)
Dimensions^14^	*✓*		*✓*	*✓*	2,184,232	Current	Daily (from 2020)
LitCovid^4^	*✓*		*✓*		422,454	Current	Ongoing daily
CoronaCentral^5^	*✓*	*✓*	*✓*	*✓*	463,294	Achieved	Final Version: Sept 2023

Earlier investigations documenting literature trends relied on citation pattern analysis and bibliographic metrics^10,11^ while more recent studies have incorporated artificial intelligence approaches to source and categorize the literature. Using the CORD-19 dataset, Mifrah and Benlahmar^12^ examined topic modelling coherence by comparing Latent Dirichlet Allocation (LDA) and Non-Negative Matrix Factors (NMF) in a corpus of 13,000 citations (published between 2019-2020). They found that the LDA probabilistic approach performed better in generating coherence of topics when texts were longer. Similarly, Bras *et. al*.^13^ retrieved 17,015 publications from the Dimensions COVID research dataset^14^ and used LDA topic modelling to rationalise the publications into a smaller hierarchical set of themes and semantic overviews. Their analysis revealed the temporal association of themes such as social distancing, mental health, and evolving trends in COVID-19 pathology, highlighting the need to develop rapid and easy interrogation of large volumes of literature. Wang and Hong^15^ extracted medical subheadings from the title and abstract of 27,370 articles from PubMed published from January-July 2020 and used Excel 2010, Medical Text Indexer (MTI), VOSviewer, and D3.js to summarize bibliometric features and to perform analysis of topics, collaboration networks, and research trends in the COVID-19 literature. Porter *et. al*.^16^ blended bibliometric and text mining methods to profile the research literature. They probed and reassembled COVID-19 topics that addressed research related to the COVID-19 pandemic. Dong *et. al*.^17^ extracted 35,092 coronavirus-related articles published up to March 20, 2022. They trained a topic model from the corpus, analyzed the semantic relationships between topics, and compared the topic distribution between COVID-19 and other SARS-CoV-2 infections. The work by Bras *et. al*. and Dong *et. al*. were limited to medical research publication platforms only. The dataset and the data processing pipeline presented in this paper leverage the open metadata from Crossref.org and DataCite.org, creating a large cross-disciplinary literature dataset. We have enriched this information using the ResearchGraph.org network to complement some of the missing metadata. This new dataset has an improved and more comprehensive coverage of papers, individually tagged with related topics, and includes more extensive metadata. In compare to the other related work (Table 1), the presented dataset in this paper expands beyond COVID 19, and encompasses twenty years of research in Coronaviruses.

The primary contributions of this work are summarised as follows. **Comprehensive Coronavirus Research Dataset**: This paper presents an open-access dataset containing metadata spanning over 20 years of research on coronaviruses, including but not limited to COVID-19. This dataset aggregates global research articles into thematic clusters, facilitating systematic review and analysis. **Enhanced Metadata Quality**: The provided metadata is meticulously cleaned, removing machine-generated tags such as HTML, ensuring higher data quality and usability for subsequent analyses. **Scalable and Accessible Trend Analysis**: This dataset features results from a scalable unsupervised topic modeling method designed to identify major research topics and explore trends over time. By examining the chronological order of these clusters, the dataset offers insights into the evolving landscape of coronavirus research before and after the pandemic.

## Methods

**Fig. 1:**
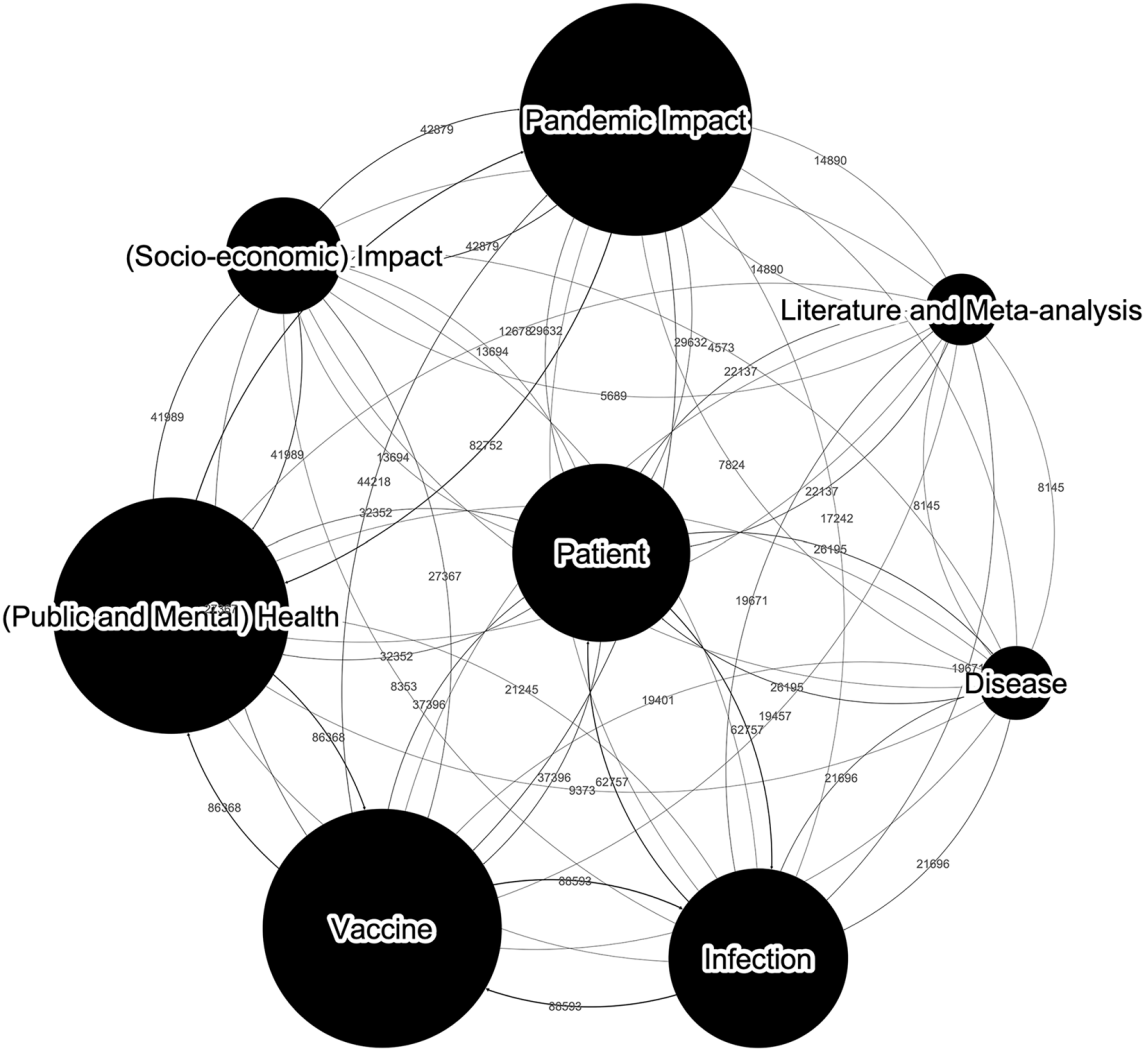
Connected graph of Coronavirus research topics (2002-2024). The size of the nodes represents the number of papers with the highest weight allocation to the cluster, and the strength of the edge represents the co-occurrence between clusters.

**Fig. 2:**
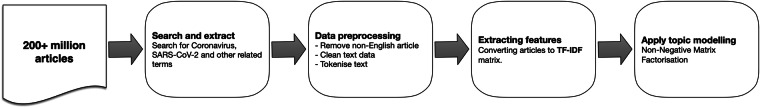
The overview of the data pipeline.

In this work, we have adopted a mixed approach that combines NLP and expert knowledge. Our methodology enabled us to scan a large number of articles (more than 200 million), identify papers related to SARS-CoV-2, and extract a matrix of interconnected topics broadly related and relevant to SARS-CoV-2 research, impact, and clinical management. Our approach demonstrated the emergence and new focus on a range of topics, including *Pandemic Impact* and *Mental health*, along with established research trends such as *Vaccine* and *Infection*. Figure 1 demonstrates the interconnectivity between these topics. In this section, we will describe how this dataset has been extracted from the extant literature, and how we have adopted a hybrid approach, augmenting topic modelling (a form of natural language processing) with domain expert review to extract, tag, and connect topics provided in this dataset. Figure 2 provides an overview of our data pipeline as we have searched, identified, extracted, and analyzed literature metadata.

- Applying text search for terms such as *COVID* and *Coronavirus* in titles, keywords, and abstracts of the papers to identify the related coronavirus papers (Section 2.2). The search results include 993,981 articles.
- Filtering non-English papers and cleaning the text to gain consistent results from topic modelling. The result of this step has reduced the number of articles to 828,566. The non-English articles were removed because earlier analysis showed multilingual text has a significant negative impact on NMF clustering. To provide consistent results, we limited our work to English articles.
- Tokenising the text by transforming text to terms consumable by the topic modelling process.
- Creating a Term Frequency - Inverse Document Frequency (TF-IDF) matrix from the tokenised text as described in Section 2.4.
- Applying topic modelling to create clusters of articles, and using a domain expert review panel to identify the best human-readable description for the clusters.

### Data lineage

This work was initiated by the Research Graph Foundation (https://researchgraph.org), following the earlier developments by the Research Data Alliance Working group on Data Description Registries Interoperability. An open data collection by this group was published by Nature Scientific Data in 2018, which made their work publicly accessible^18^. Research Graph has adopted the Research Data Alliance Working Group outcome, and leveraged their proposed graph model to provide the capability for this project. The graph model uses the interconnectivity between research objects (publications, grants, datasets) across scholarly networks to gain insights into the latest research. In late 2020, the foundation initiated the COVID19-Graph project, which benefited from the collaboration of researchers in multiple universities and government agencies in Australia. This open-access dataset is the outcome of this work containing the classification of 828,566 papers. Using Research Graph Augment API, this project has identified related papers and enriched the metadata of publication records by extracting related fields from crossref.org, datacite.org, and pubmed.ncbi.nlm.nih.gov.

### Search and extract

Building upon the open Research Graph platform, we have established a comprehensive and reproducible pipeline to systematically extract research articles that contain the terms *COVID*, *Coronavirus*, and *sars-cov-2* in their titles, abstracts, and keywords. Our methodology involves leveraging several open scholarly metadata repositories, including Crossref, DataCite, and PubMed, to ensure a broad and inclusive search. Additionally, we access various open-access repositories through the Research Graph network to further expand our dataset.

The scope of our search encompasses an extensive collection of 160,374,530 metadata records, each identified by a digital object identifier (DOI) including journal articles, conference papers, reports, books, dissertation and other scholarly outputs such as datasets. The search results have yielded 993,981 records. From this vast dataset, we have meticulously extracted metadata, capturing crucial attributes such as the title, abstract, publisher, subjects, authors, and the creation date of the articles. This comprehensive metadata extraction allows for a detailed and nuanced analysis of the research landscape related to COVID-19, facilitating further studies and insights into the global research efforts on the pandemic.

**Fig. 3:**
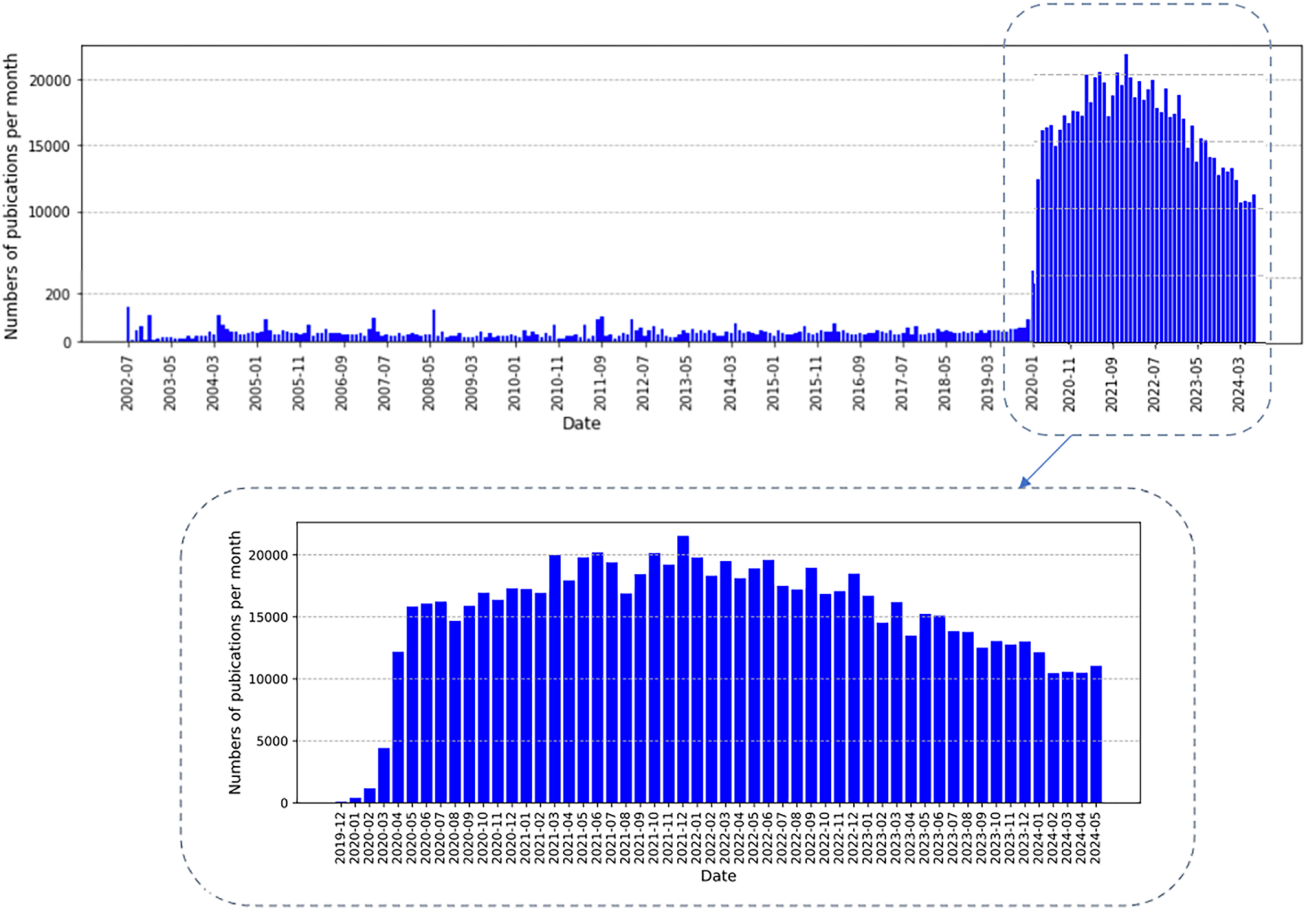
The number of coronavirus-themed articles published between July 2002 and May 2024.

Figure 3 illustrates the number of coronavirus-themed articles published on a monthly basis between July 2002 and May 2024. This figure highlights the research activity within this period, showcasing a significant escalation in publications. Notably, research activity increased exponentially following the identification of COVID-19-positive cases, leading up to the World Health Organisation’s formal confirmation of the pandemic in early 2020. As a result of this heightened research focus, the number of publications surged dramatically, reaching a peak of over twenty thousand articles being published monthly during the pandemic’s most critical phases.

### Data pre-processing

To run text mining on research topics, the first step is to extract the title from each article and run pre-processing on the titles including the following steps.

- Remove non-English articles in the datasets, then filtered out extra spaces, HTML tags, section headings, punctuation, and special characters.
- All uppercase characters were converted to lowercase because the form of words can affect topic modelling algorithms.
- Applying tokenization using text segmentation tools, such as the NLTK tokenizer (nltk.org), to split text into words (tokens).
- Applying the lemmatization method^19^ to group inflected forms of a word, allowing them to be analyzed as a single item.
- Removal of stopwords, which are words commonly encountered without dependency to a particular topic (e.g. 'it', 'am', 'the'). In addition, we removed the following high-frequency words that do not facilitate topic grouping including 'covid', 'sars', 'study', 'learning', 'student', 'online', 'education', 'case', 'analysis', 'coronavirus'. These stop words and high-frequency terms are confirmed by a nominal expert panel to remove, leading to better clustering results (Section 2.6).

Once the text corpus was established, we analyzed the data by extracting features, applying topic modelling, and creating visualizations. The following sections further expand on the data analysis and transformation processes used.

### Extracting features

The Term Frequency - Inverse Document Frequency (TF-IDF) is a statistical measure used to extract keywords from a collection of documents. It is calculated by multiplying the number of times a word appears in a document (term frequency) by the inverse of the frequency of documents containing that word (inverse document frequency) across the entire collection. A higher TF-IDF score indicates that the word is more significant or relevant to the document. For example, in our collection, words like ‘patient’, ‘pandemic’, ‘disease’, and ‘health’ appear in most documents; therefore, these words have lower TF-IDF scores despite their frequent occurrence. In our work, TF-IDF scores are the main input for the topic modelling process.

### Topic modelling

To understand and categorize the publications, we have leveraged topic modeling tools^20^. Topic modeling extracts clusters or categories automatically from unlabeled documents in an unsupervised process.

Non-negative matrix factorization (NMF) is a widely used technique for high-dimensional data analysis. The algorithm follows the distributional hypothesis, as discussed by Sahlgren^21^. NMF decomposes the original corpus, represented by a Document-Term Matrix, into two smaller matrices. This decomposition is useful for dimensionality reduction, source separation, and topic extraction.

**Fig. 4:**
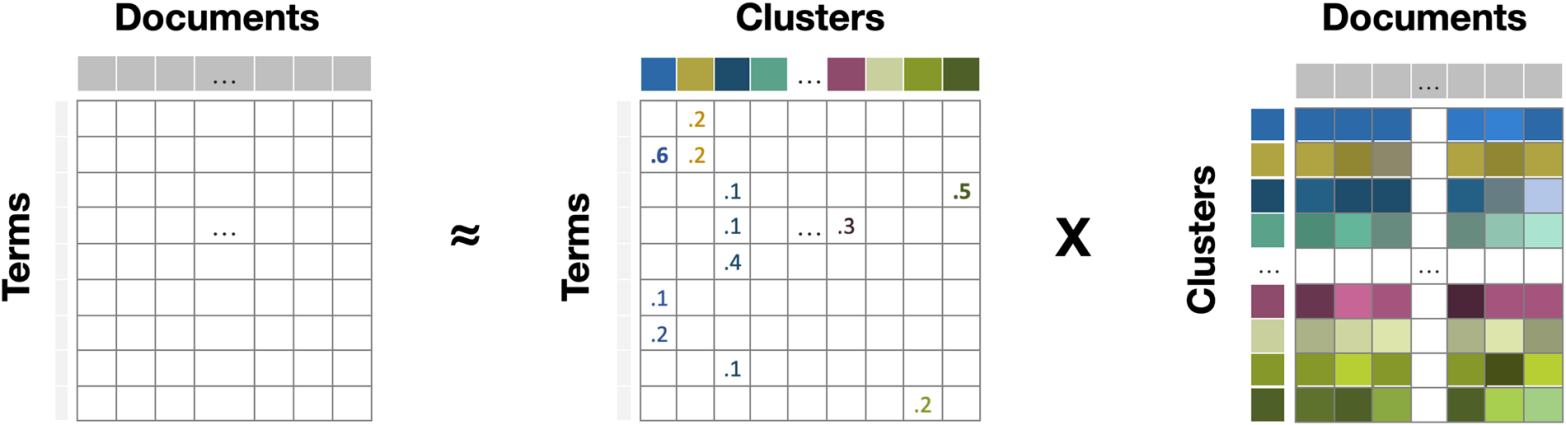
An illustration of the topic modelling process by NMF.

As illustrated in Fig. 4, the Document-Term Matrix (M) is decomposed into a matrix of (un-normalized) probability cluster weights for each document and a matrix containing coefficients of terms for each cluster^22^. Unlike other algorithms, NMF avoids the sum-to-one constraints on the cluster weights, resulting in a simpler optimization problem and more efficient computations^23^. This process assigns each document to clusters with weight values representing the strength of the connection.

**Fig. 5:**
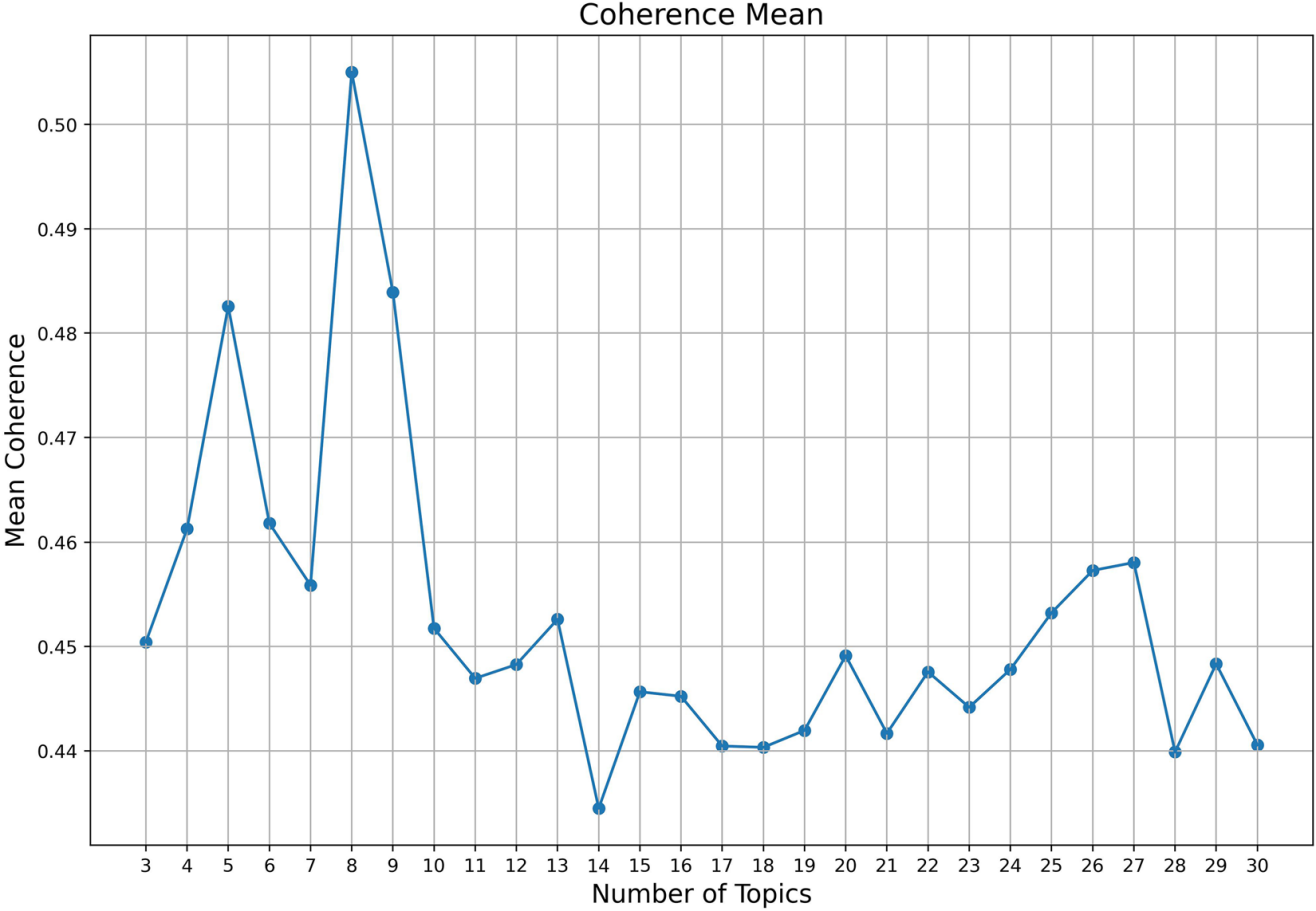
Mean of coherence scores between top 5 keywords in topic size 3 to 30.

A prior determination of the number of clusters is required as a parameter for the NMF algorithm. While there is no standard approach for selecting this number^24^, various methodologies have been considered due to their significant impact on the quality of topic modelling results. A widely recognized solution is to combine both quantitative analysis and expert-based Collaborative analysis. We have applied coherence score^25^ to evaluate the quality of topics generated. The coherence score reflects the relevance between terms within a topic, which, based upon the distributional hypothesis^21^, reflects semantic consistency and, hence, the quality of topics. We used the top 5 terms to compute the coherence scores for clusters between three and thirty. The mean of coherence scores for topics are shown in Fig. 5. The peaks of coherence scores appeared around a cluster scale of three and eight. Reviewing the results by the research team revealed that eight was the biggest number of clusters that produced a high coherence level while providing meaningful separation of topics.

**Fig. 6:**
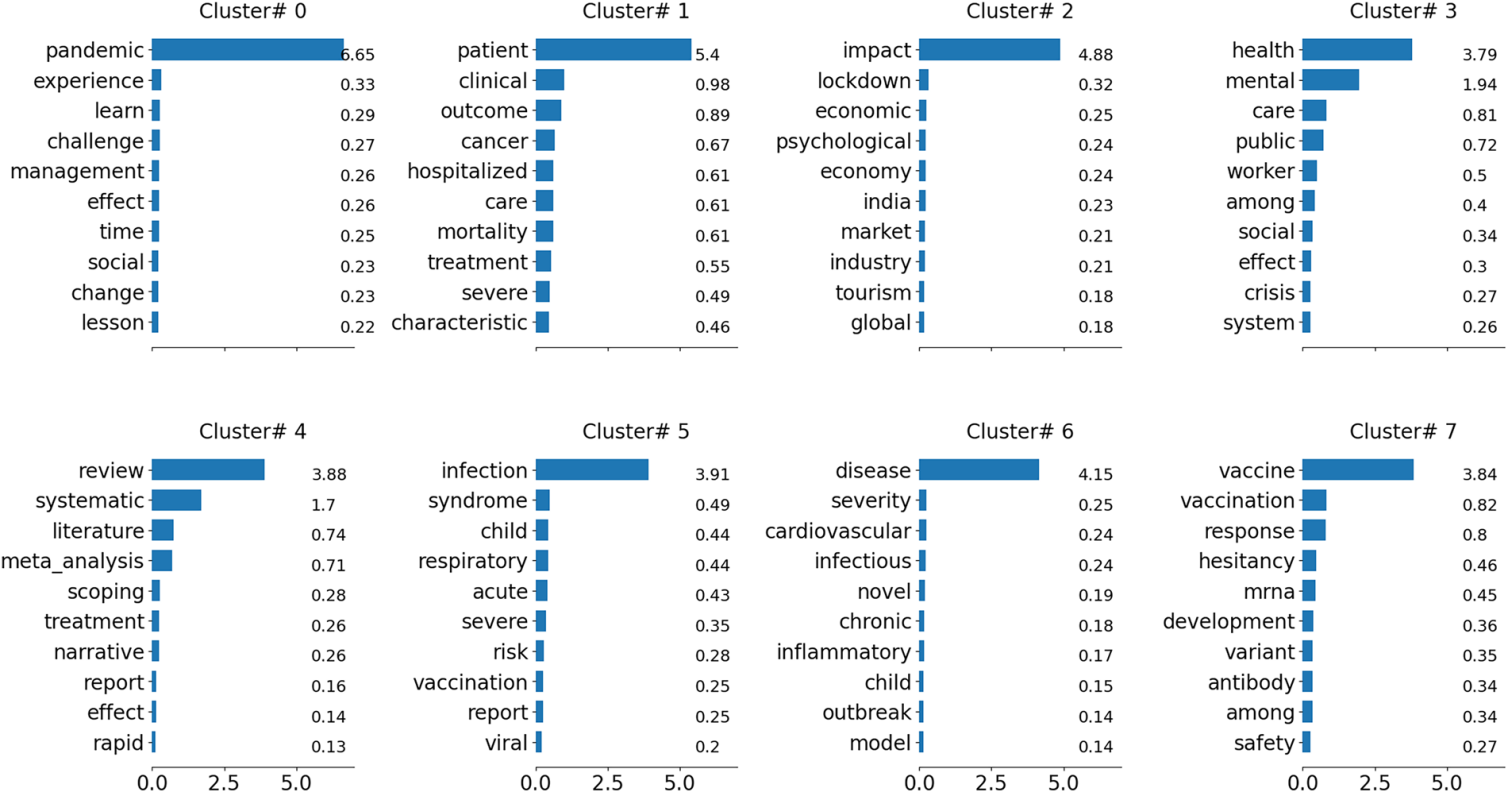
Top 10 keywords for clusters.

Figure 6 shows the top-weighted keywords for each cluster sorted by the maximum weight value assigned by the NMF algorithm. Most clusters have one or two dominant keywords that describe the main topic in the cluster. The next section will discuss how these keywords align with the health experts’ recommended topics for each cluster.

### Expert panel review

We utilised nominal expert panel review^26^ to evaluate how clusters are forming and allocating papers to appropriate research topics. The panel reviewed the results as part of the post-process quality control step. The panel comprised academics and scientists trained in biology, medical science, public health, digital health, computational social science, and mental health. You will find the panel as the co-authors of this paper.

The review process involved several key steps:

- Preparatory Meeting: The panel members participated in an initial orientation session to ensure a common understanding of the review criteria and procedures. The objectives of this review were described as: firstly, evaluating the connections between clusters and articles; secondly, reviewing the clusters’ overall topics and coherence; and finally, reviewing the stop words and enabling an iterative process for improving the results by refining the stop words.
- Paper Selection: We selected sixty papers from each cluster using a stratified random sampling method to ensure a representative subset of the cluster’s content. Each panel member received 30 papers, marked with the two clusters with the highest weight (Section 2.5).
- Review Tools and Resources: Panel members were provided with access to full publication metadata and the full text of the articles. They utilised a custom annotation tool designed to facilitate the review process.
- Evaluation Metrics: Experts assessed the papers based on relevance, coherence, and topic overlap. This was a qualitative exercise, as most panel members took notes on papers and related topics, providing ample material for discussion.
- Reviews and Discussions: Following the individual reviews, we conducted four iterations of group discussions to reconcile differences and integrate feedback. During these discussions, panel members reviewed the results and discussed the assigned topics. Additionally, the members reviewed the stop words (Section 2.3) and provided suggestions for variations of these stop words. The panel also reviewed the top keywords for each cluster and recommended titles for these clusters. Table 2 summarises the top five keywords in each cluster, and the recommended topic by the expert panel (Section 2.6).

**Table 2:** Cluster generated by NMF topic modelling, top keywords, and recommended topics by domain experts.

Cluster	Keywords	Recommended Topics
# 0	pandemic, experience, learn, challenge, management	Pandemic
# 1	patient, clinical, outcome, cancer, hospitalized	Patient
# 2	impact, lockdown, economic, psychological, economy	(Socio-economic) impact
# 3	health, mental, care, public, worker	(Public and Mental) Health
# 4	review, systematic, literature, meta-analysis, scoping	Literature and meta-analysis
# 5	infection, syndrome, child, respiratory, acute	Infection
# 6	disease, severity, cardiovascular, infectious, novel	Disease
# 7	vaccine, vaccination, response, hesitancy, mRNA	Vaccine
- Iteration Process: We incorporated the comments and feedback from the panel into the NMF process. Two major changes to the NMF parameters were testing alternative clustering numbers and incorporating new stop words. We reran the NMF algorithm multiple times with different parameters and evaluated the coherence scores (Fig. 5), distribution of top keywords (Fig. 6), and co-occurrence of the articles (Fig. 9), as described later in technical validation (Section 4.1).
- Major Findings: Following the iterative process of running NMF guided by the nominal expert panel feedback, we identified the following major findings: Firstly, the nominal expert panel confirmed that the optimum number of clusters identified by the mean of coherence scores for topics (Fig. 5) is the best option, as dividing articles into fewer or more clusters introduces more overlaps between clusters and creates ambiguity between the connection between articles and related topics. Secondly, the panel introduced new stop words that effectively reduced the inconsistency between the top keywords in each cluster (Fig. 6) and improved the clustering consistency. The final agreed stop words by the panel are listed in Data Pre-processing (Section 2.3). Finally, the panel’s strong agreement with the coherence of the NMF clustering results confirmed the usefulness of this clustering method for large-scale knowledge mining from literature, leading to discussions about its promising potential for other health-related topics.

Table 3 presents some of the papers reviewed by the panel. While this table represents only the title and publication date, the panel had access to full publication metadata and full text of the article. It is important to emphasise that since the panel reviewed only a few papers, the review results were solely utilised to further understand the clustering outcomes. The review’s scope is too narrow to be considered a systematic assessment of the topic modelling output. We propose that utilising published open-source code 6 would be a more reliable method of evaluating our clustering approach.

**Table 3:** Example articles for each cluster.

	Publications
Cluster# 0	COVID-19 Pandemic, Materials Horizons: From Nature to Nanomaterials, Jan 22,^37^
	How COVID pandemic may end : Co-existence is the key, Asian Journal of Medical Sciences, Feb 22,^38^
	The effects of the pandemic, Early Years Educator, May 22,^39^
	The U.S. Response to the COVID-19 Pandemic, Policy Styles and Trust in the Age of Pandemics, Feb 22,^40^
	What we are learning from the COVID-19 pandemic, Journal of American Association for Pediatric Ophthalmology and Strabismus, Aug 22,^41^
Cluster# 1	Xerostomia: What to Do in COVID-19 Patients, International Journal of Basic Science in Medicine, May 22,^42^
	Analysis of COVID-19 Patient Follow-Ups, Life and Medical Sciences, Jan 22,^43^
	COVID-19 in cancer patients, Transactions of The Royal Society of Tropical Medicine and Hygiene, Mar 22,^44^
	547 Clinical Outcomes for Burned Patients with Covid-19, Journal of Burn Care & Research, Mar 22,^45^
	Case Study of Patients with HIV during the COVID-19 Pandemic, Journal of Pure and Applied Microbiology, Feb 22,^46^
Cluster# 2	The Impact of COVID-19 on Disneyland, Advances in Economics, Business and Management Research, Jan 22,^47^
	Economic impacts of COVID-19, Map of the Month, Apr 22,^48^
	Impact of Covid-19 on the environment, Catrina: The International Journal of Environmental Sciences, Jul 22,^49^
	Impact Of The Coronavirus (Covid-19) Pandemic On Education, European Proceedings of Social and Behavioural Sciences, Feb 22,^50^
	Impact of Covid-19 on IT Company, International Journal for Research in Applied Science and Engineering Technology, Apr 22,^51^
Cluster# 3	Mental health after covid-19, BMJ, Feb 22,^52^
	On Covid-19 and mental health, Medicine, May 22,^53^
	Effect of COVID-19 on Mental Health of Health Workers, Pharmaceutical Research International, Dec 21,^54^
	Stress and Mental Health, Advances in Higher Education and Professional Development, Jun 22,^55^
	The future of public health, British Journal of Hospital Medicine, Jun 22,^56^
Cluster# 4	A Review on COVID-19 Pandemic, Journal of Clinical and Diagnostic Research, Sept 22,^57^
	COVID-19 in Carceral Systems: A Review, Annual Review of Criminology, Jan 23,^58^
	A Systematic Review on the Impact of COVID-19 to Communities, International Journal of Academic Research in Business and Social Sciences, Apr 22,^59^
	Covid and Diabetes: A Literature Review, Saudi Journal of Medicine, Sep 22,^60^
	Characteristics of Living Systematic Review for COVID-19, Clinical Epidemiology, Aug 22,^61^
Cluster# 5	Coronavirus: Infection of Piglets and Men, Journal of Medicine and HealthCare, Apr 22,^62^
	Infection Control and COVID-19, Anesthesia Student Survival Guide, Jan 22,^63^
	NAD+ in COVID-19 and viral infections, Trends in Immunology, Apr 22,^64^
	COVID-19 infections in infants, Scientific Reports, May 22,^65^
	COVID-19 Infection: Its Lingering Symptoms in Adults, Cureus, May 22,^66^
Cluster# 6	COVID-19 and Kidney Disease, Annual Review of Medicine, Jan 23,^67^
	COVID-19 and liver disease, Gut, Jun 22,^68^
	The Impact of COVID-19 on Surgical Disease, Frontiers of COVID-19, Jan 22,^69^
	Immune system aging and the aging-related diseases in the COVID-19 era, Immunology Letters, Mar 22,^70^
	The Relationship Between Inflammatory Indicators and the Severity of the Disease in Coronavirus Disease, Meandros Medical and Dental Journal, Jun 22,^71^
Cluster# 7	COVID-19 vaccine: what are we doing and what should we do?, The Lancet Infectious Diseases, May 22,^72^
	COVID-19 vaccine hesitancy, Nature Reviews Nephrology, Apr 22,^73^
	Vaccine technologies used to develop COVID-19 vaccines, Microbiology Australia, Apr 22,^74^
	Theory of COVID-19 vaccines, International journal of health sciences, May 22,^75^
	What next for covid-19 vaccines?, BMJ, Oct 22,^76^

## Data Records

**Fig. 7:**
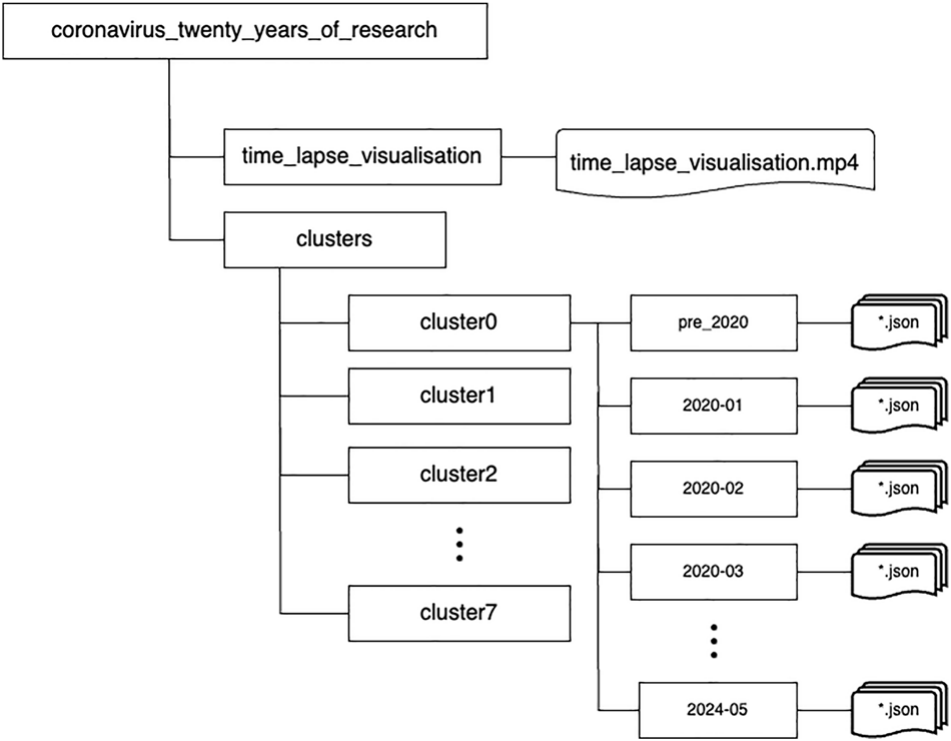
The dataset files and folders’ structure.

This dataset is available at Figshare (10.6084/m9.figshare.26940958)^27^. The Fig. 7 illustrates the dataset structure. The ‘coronavirus_twenty_years_of_research’ contains two folders. The ‘clusters’ folder is the core of our dataset that contains the classified articles into the eight clusters (Section 2.5). Finally, ‘time_lapse_visualization’ folder contains a video that animates the research trends (Section 4.3).

**Fig. 8:**
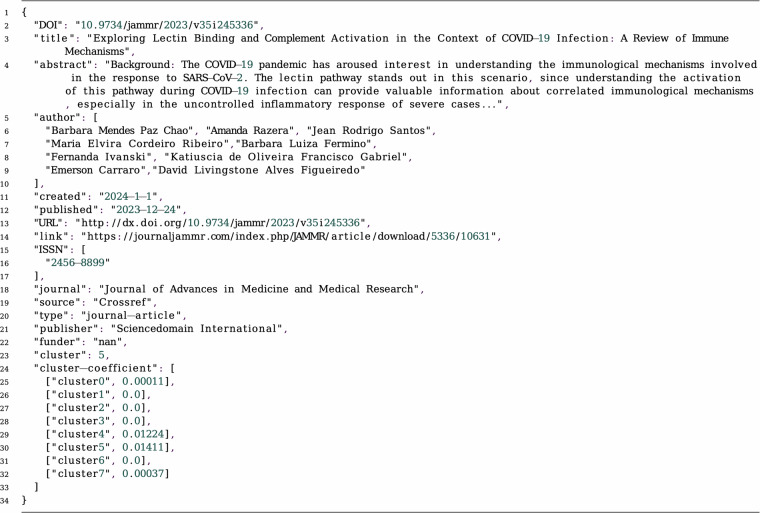
Metadata fields of a publication JSON file (Note: Abstract reduced to initial sentences for printing).

The metadata files are organised based on their creation date. The majority of articles are published post 2020, hence, we have collected all earlier papers in ‘pre_2020’ folder. Publication from 2020 are collected per month in folders labeled ‘YYYY-MM’. Each JSON file includes a single publication record and is named based on the article’s DOI. To make the file names compatible with UNIX/MAC Windows operating systems, we have replaced the slash(‘/’) with a hyphen(‘-’). For example, for the following publication: “Mohapatra, M., Choudhury, B. & Basu, S. (eds.) COVID-19 Pandemic (Springer Nature Singapore, 2022), 10.1007/978-981-16-4372-9” is published in Jan 2022 you will find the JSON file at ‘dataset/2022-01/10.1007-978-981-16-4372-9.json’. Figure 8 shows an example of the JSON files, and the description of the metadata fields is available in Table 4. This metadata schema is interoperable with Crossref Schema version 5.3.1^28^.

**Table 4:** Description of metadata fields.

Field name	Description
DOI	Unique identifier for each individual publication
title	Title of the publication
abstract	Abstract of the publication (limited to open access publications)
author	Author(s) of the publication
created	The date when the publication is created
published	The date when the publication is published
URL	A web link to a publisher’s homepage
link	Information including Uniform Resource Locator of the publication
ISSN	The International Standard Serial Number assigned to the title being registered
journal	The title of the journal where the paper is published
source	The source of the publication
type	The type of the publication
publisher	An organization that published the paper
funder	A provider of funds
cluster	Cluster(s) of the research from NMF topic modelling
cluster-coefficient	The membership weights for the topics

Important Note: The availability of the “Abstract” property depends on the article’s open access status. We verified this field using the Crossref API, and only abstracts available through the Crossref Open Access API are included. This limitation is due to copyright restrictions.

## Technical Validation

This section presents an analysis of the dataset content, detailing the characteristics of the clusters and offering insights into the technical quality of the records. Our analysis includes four main components: Firstly, we evaluated the coupling and overlap between clusters (Section 4.1). This evaluation provides a deeper understanding of how closely related the clusters are and the extent to which they share common features. Next, we conducted an analysis of key metadata fields, including Publisher, Journals, and Funders, across all clusters (Section 4.2). This analysis sheds light on the distribution of articles, revealing patterns and trends that contribute to understanding the types of publications in each cluster. We also examined the evolutionary trends of these clusters (Section 4.3). By analyzing how the clusters have developed over time, we can identify emerging themes and shifts in research focus, providing context for understanding the current state and future directions of the Coronavirus research. Finally, we employed generative AI to evaluate the outcomes of the topic model results (Section 4.4). This technique allowed us to assess the coherence and relevance of the topics generated, ensuring that the model’s findings align with the topic modeling results.

### Relation between topics

One of the outputs of NMF topic modelling is a coefficient matrix that connects documents to clusters based on the coefficient weight value^21^. While we have assigned each article to the most probable cluster (cluster with the highest weight value), one could consider each article to be linked to multiple clusters with different priorities based on the strength of the weight value.

For each pair of clusters i and j, the co-occurrence was calculated based on the number of publications in either of the clusters and the proportion of publications having the second highest weight in the other cluster, i.e., the co-occurrence between clusters i and j is calculated by

$${C}_{i,j}=\frac{{N}_{i,j}+{N}_{j,i}}{{\sum }_{t=1}^{8}{N}_{i,t}+{N}_{j,t}}$$

where *N*_*i*,*j*_ presents the number of articles having the highest two weights for clusters i and j, respectively.

**Fig. 9:**
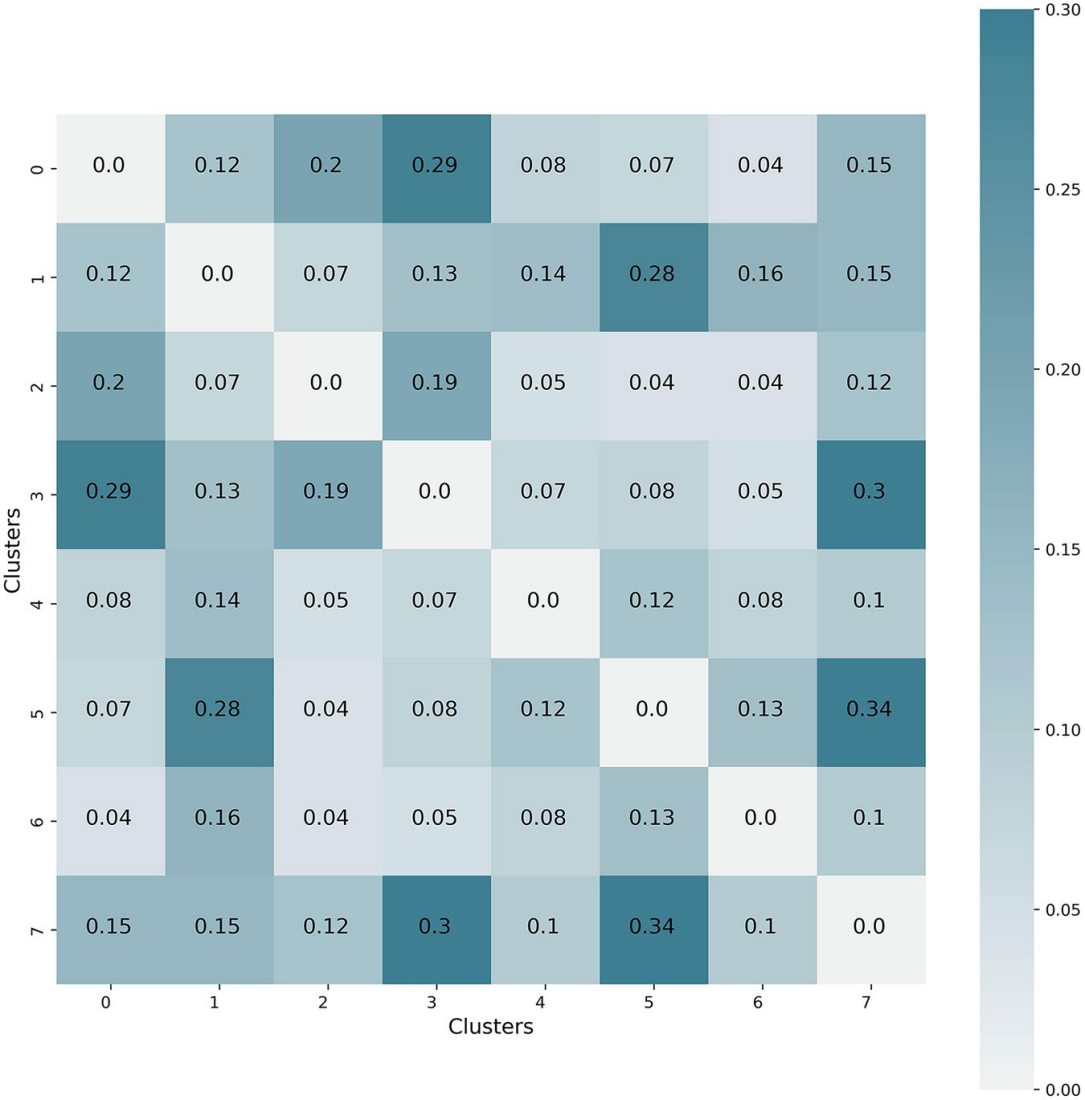
Articles co-occurrence rate over the clusters. **Cluster 0**: “Pandemic”, **Cluster 1**: “Patient”, **Cluster 2**: “(Socio-economic) Impact”, **Cluster 3**: “(Public and Mental) Health”, **Cluster 4**: “Literature and Meta-analysis”, **Cluster 5**: “Infection”, **Cluster 6**: “Disease”, **Cluster 7**: “Vaccine”.

Figure 9 presents the co-occurrence rates across the eight identified clusters in the form of a matrix. Notably, the highest co-occurrence rate, recorded at 0.34, is observed between clusters five and seven. This indicates a significant interrelation between articles pertaining to “Infection” and those concerning “Vaccine”. In contrast, cluster two exhibits the lowest co-occurrence rates with clusters five and six, both at 0.04. This shows a stark contrast between articles discussing the “Socio-economic impact” of the pandemic and those focused on “Infection” and “Disease”. A similar pattern is observed between clusters zero and six, further underscoring the distinct separation between studies exploring the societal impacts of the pandemic and those addressing medical topics such as infection syndromes and cardiovascular diseases.

It is important to note that these variations in co-occurrence rates reflect the distinct thematic boundaries of the clusters. The low co-occurrence rate between clusters focusing on socio-economic impacts and those on medical issues highlights the diversity of research topics in the dataset. This separation is crucial for understanding the different dimensions of pandemic-related research. Moreover, the relatively low co-occurrence rates across all clusters suggest that the clustering algorithm has effectively grouped articles into categories with minimal overlap, enhancing the clarity of thematic distinctions. In summary, the co-occurrence rates do not exceed 0.35 across any clusters, indicating only weak inter-cluster coupling. This low co-occurrence rate across clusters suggests that the clustering results are robust, effectively segregating articles into well-defined thematic groups. These findings demonstrate the efficiency of the clustering process in distinguishing between various research areas, thereby providing a clear and organized structure for analyzing the literature on pandemic-related topics.

### Publication metadata

**Fig. 10:**
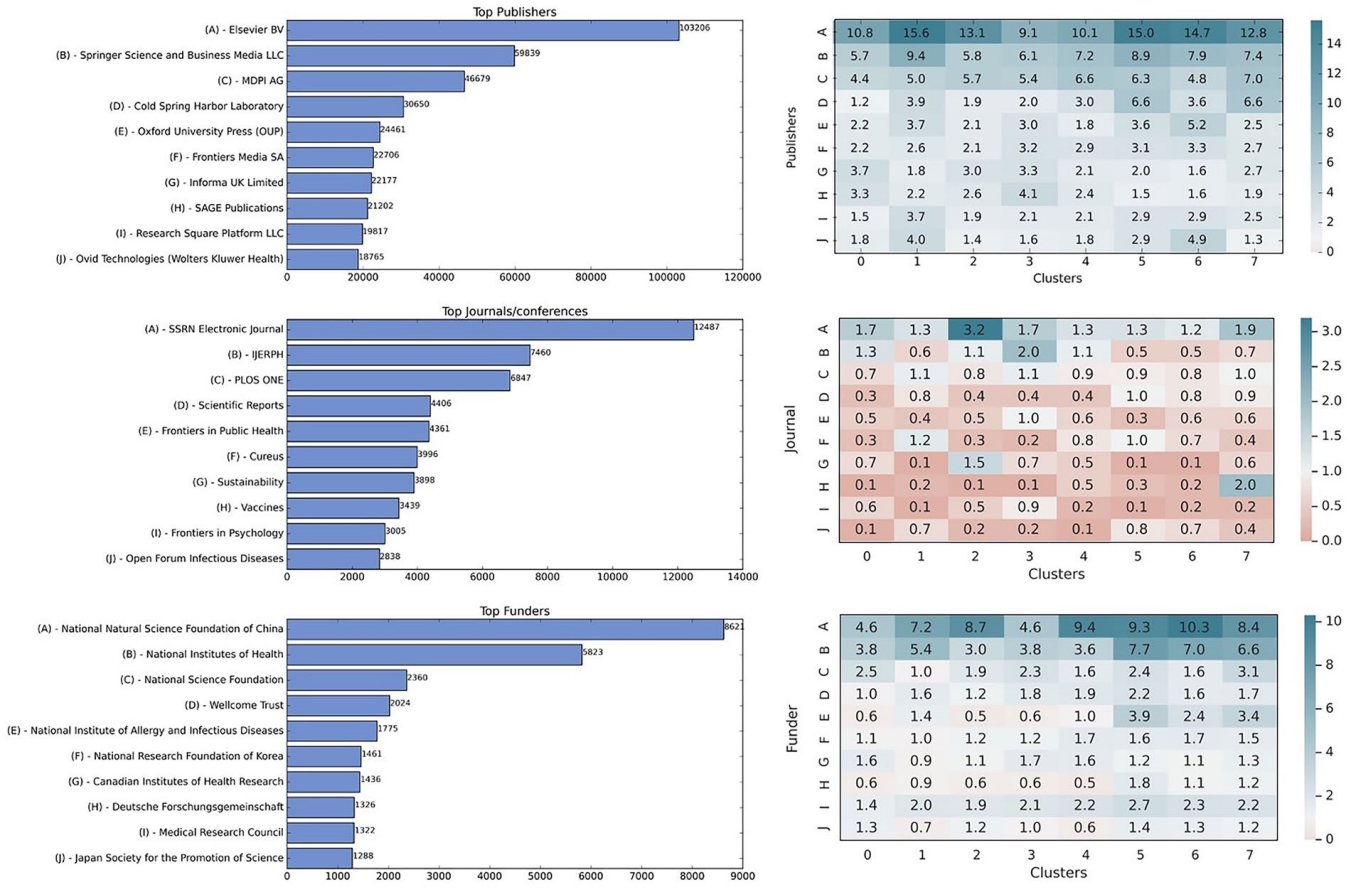
The overview of the publications’ metadata.

The COVID literature dataset compiled in this study includes a rich set of metadata for publications. Figure 10 displays major categories (Publishers, Journals, and Funders) and the percentage of publications within each cluster. This figure displays only the top ten items in each category.

**Publisher:** 828,531 papers have publisher information (99%), with 13,544 unique publisher values. Elsevier leads in publication volume, contributing 103,206 articles across all clusters, presented in the left bar chart. The right-hand tables in Fig. 10 detail the proportion of articles published by Elsevier within each cluster. In Cluster#0 and Cluster#1, Elsevier published 10.8% and 15.6% of the articles, respectively (as shown in the table on the right side of Fig. 10).

**Journals:** This field lists the name of the journal or conference name, and across this dataset 88% of articles have a value for this field, totaling 728,020 articles. SSRN Electornic Journal is the most common publishing target for all clusters, except Cluster#3 where the International Journal of Environmental Research and Public Health (IJERPH) marginally surpasses SSRN. However, even the top ten journals for each cluster hold only a small portion of articles (1-3%); this shows the diversity of journals and conferences in all clusters.

**Funders:** In the dataset, funding information is available for 116,424 publications, which represents 14% of the articles. It is common for a single paper to list multiple funding sources, and as a result, 62,168 unique funders have been identified. The National Natural Science Foundation of China is the most frequent sponsor, supporting more publications than any other entity, followed by the National Institutes of Health (NIH) and the National Science Foundation (NSF).

**Abstract:** One of the most sought-after metadata fields for publications is the “Abstract”. This field is particularly useful for machine learning and other data mining methods. In this dataset, we have cleaned the abstract fields by removing HTML and other machine-generated tags. We have included abstracts only when the open access license allowed us to share them, as identified using the Crossref API. In Crossref metadata, articles lacking abstracts are typically editorials, letters, news items, book reviews, older articles, short-form notes, and most commonly articles from publishers not depositing abstracts. Overall, 470,141 publications include abstracts, representing 57% of all articles.

### Distributions and trends

**Fig. 11:**
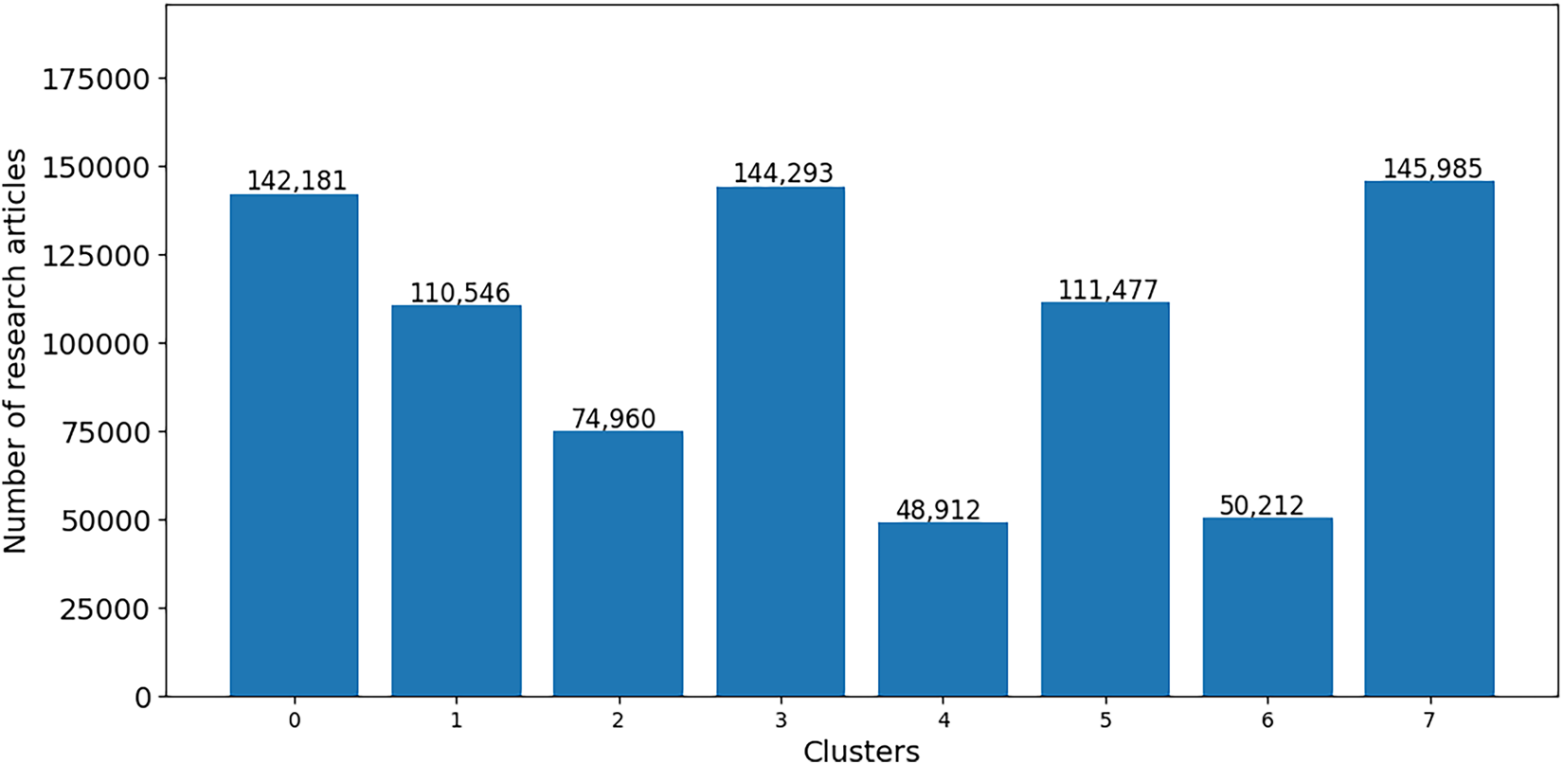
Distribution of articles across clusters.

**Fig. 12:**
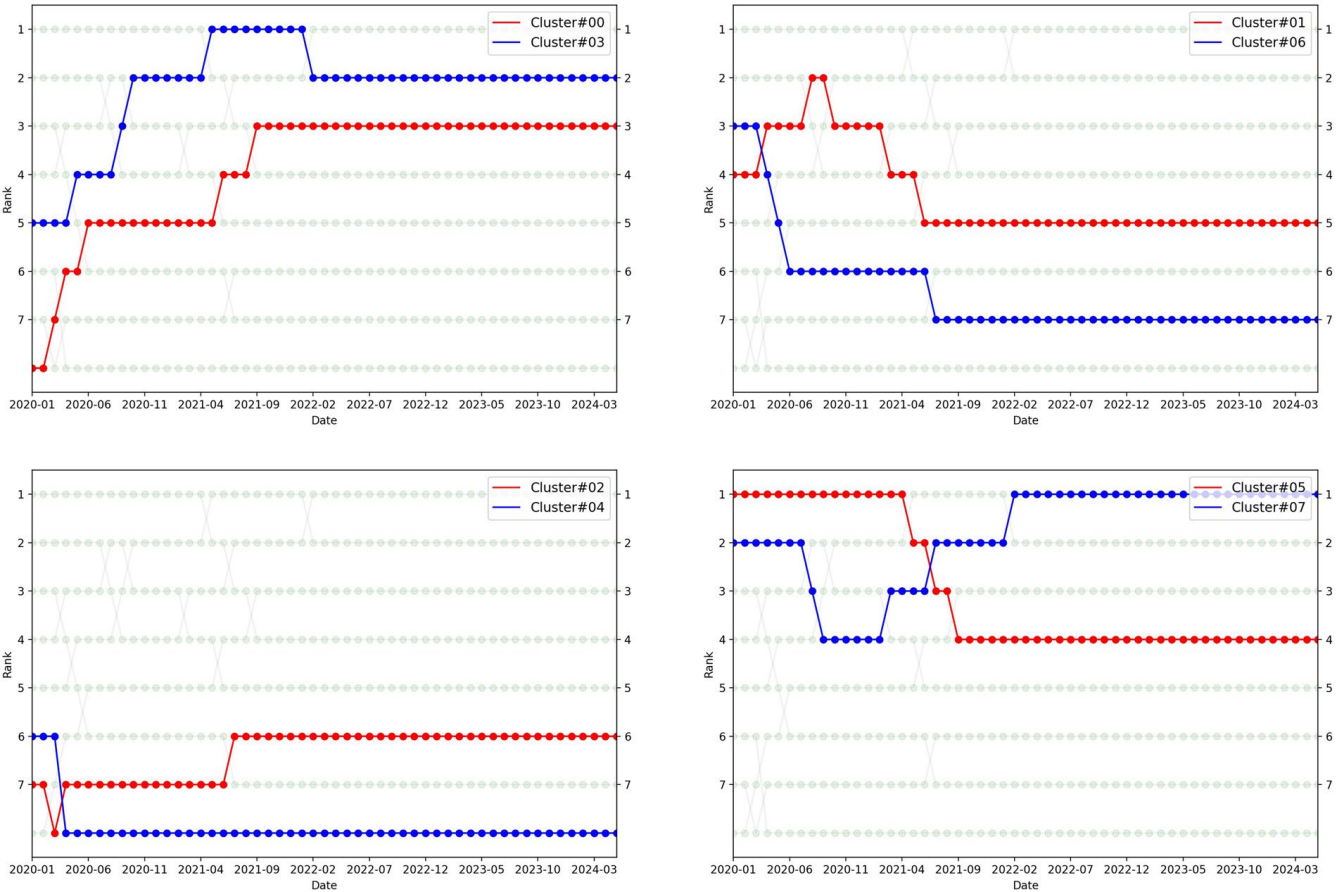
Research topic trends vary over time. **Cluster 0**: “Pandemic”, **Cluster 1**: “Patient”, **Cluster 2**: “(Socio-economic) Impact”, **Cluster 3**: “(Public and Mental) Health”, **Cluster 4**: “Literature and Meta-analysis”, **Cluster 5**: “Infection”, **Cluster 6**: “Disease”, **Cluster 7**: “Vaccine”.

As outlined in Section 2.5, we applied the topic modeling algorithm to analyse the collection of articles, forming eight clusters (labeled #0 to #7) based on their thematic content, with articles grouped according to the highest associated weight values (Fig. 11). The number of articles within these clusters varied over time. For instance, Cluster 5 (Infection) was the largest in 2019, but by mid-2021, Cluster 3 (Public and Mental Health) emerged as the most populated. This dynamic change in cluster ranking from December 2019 to May 2024 is visually represented in Fig. 12. Additionally, we have documented these changes in a time-lapse video, stored in the ‘time_lapse_visualization’ folder, which effectively captures the temporal transitions among the clusters, as depicted in Fig. 7.

### Evaluating using Large Language Models

For validation, we evaluate the results of the NMF algorithm using GPT-4 Turbo. Due to the prohibitive costs and computational demands, testing all publications involved in this analysis with GPT-4 Turbo was not feasible. We randomly selected 100 papers from each of the 8 clusters defined by NMF and used GPT-4 Turbo to evaluate whether NMF found the corresponding cluster for each article. To avoid creating bias in the LLM context, the sampled articles were processed one at a time using the following prompt. The source for this step is available at *source/Evalue-Clusters-Using-GPT4.ipynb* in the GitHub repository (described in Section 6).

The GPT-4 Turbo model processed 800 samples, requiring over 4 hours and 29 minutes to generate results. It produced probability values ranging from 0 to 0.95. A threshold of 0.5 was established; values above or equal to this threshold suggest that GPT-4 agrees with the NMF-assigned cluster, while values below 0.5 denote a discrepancy. In addition, we have compared these results with Llama 3.1(70b), a state-of-the-art open-source LLM at the time of writing this paper. Table 5 shows the results of the GPT-4 and Llama 3.1 models.

**Table 5:** Comparative Analysis of GPT, Llama 3.1, and NMF Outputs Across Different Clusters with Average Precision.

Clusters	GPT Confirmed Articles	Llama 3.1 Confirmed Articles	NMF Retrieved Articles	Avg. Precision (%)
#0	99	94	100	96.50%
#1	97	83	100	90.00%
#2	61	57	100	59.00%
#3	90	89	100	89.50%
#4	73	83	100	78.00%
#5	86	88	100	87.00%
#6	93	89	100	91.00%
#7	60	68	100	64.00%
**Grand Total**	**659**	**651**	**800**	**81.88%**

The results are promising, as the average precision for the probability of the alignment between the article and the collection is 81.88%, confirmed by GPT-4 and Llama 3.1. We measured the precision of this assignment by calculating the true positives (confirmed by LLM models) divided by the number of retrieved articles for each cluster. The highest precision was observed in cluster #0 (“Pandemic”), where 99% of the articles were confirmed by GPT-4 and 94% confirmed by Llama 3.1. In contrast, the lowest precision is 57% and 61% by Llama and GPT for cluster #2 (“Socio-Economic Impact”). These findings suggest that large language models like GPT-4 Turbo can yield results comparable to traditional topic modelling for certain topics, while results may vary for others. These results suggest that the NMF algorithm and LLM can be used as complementary tools, particularly when the time and cost of LLM computation is a barrier to large-scale knowledge mining.

### Dataset Constraints and Limitations

In any data-driven research, acknowledging the limitations of the dataset is crucial for understanding the scope and applicability of the findings. This section outlines key constraints associated with our dataset and methodology, providing context for interpreting the results and identifying areas for future improvement.

The classification approach we employed is coarse-grained, designed to illustrate general research trends rather than capture the detailed nuances of specific topics. This method provides an overview of major research streams but lacks the granularity needed to map to precise medical or socio-economic taxonomies. To achieve more detailed mappings, other classification methods such as machine learning can be employed, that are often more computationally expensive. One of the advantage of the current method is its ability to reduce the number of articles required for such methods leading to more efficient hybrid knowledge mining pipelines.

While abstract of the articles is an essential information field for many knowledge mining activities such as machine learning, in this dataset we can only shared the abstracts of open-access articles, excluding abstract content for articles behind paywalls. This restriction means that the dataset may not fully represent all relevant research and perspectives. This is a limitation imposed by the copyright restrictions.

Furthermore, the scale of the dataset poses challenges for expert review; the limited scope of papers reviewed by experts suggests that alternative evaluation methods might offer more accurate insights into classification accuracy. We provided the result of our experiment with the validation of NMF clustering using large language models (LLMs); however, there is significant potential to enhance clustering methods through more direct application of LLMs. Although the high computational cost associated with fine-tuning or running large LLMs on a dataset of this size currently limits our ability to fully exploit these models, we believe that future research may uncover new ways to leverage LLMs for large-scale clustering or evaluation of existing clusters such as this dataset.

## Usage Notes

The literature on SARS-CoV-2 and the COVID-19 global pandemic rapidly grew in early 2020. Our analysis indicated that by March 2021, approximately 20,000 COVID-related articles were published monthly. While the frequency of publications has declined, the volume of articles remains significant. Despite platforms such as CORD-19, and the WHO and CDC COVID-19 publication datasets no longer being updated, seasonal variations in infection frequency and the emergence of health-related issues including long COVID^29^, and cardiovascular^9,30^ and neurological complications^31^ of COVID-19 render the topic of ongoing importance.

Using publications derived from the WHO COVID-19 dataset, Pal (*et al*.)^32^ demonstrated the utility of machine learning approaches to predict themes in the COVID-19 literature. Our analysis, using topic modelling tools to annotate over eight hundred thousand articles, demonstrated clear temporal trends in the frequency of publication themes (Section 4.3). These trends could be used in near real-time to monitor and guide policy and resource allocation. Indeed, our data pipeline and literature dataset can provide a valuable resource for those focused on the impact of COVID-19. It contains vast information covering various aspects of the pandemic, including epidemiology, clinical research, public health interventions, and economic impact. Researchers, academics, policymakers, and healthcare professionals can use this dataset to explore COVID-19 research findings, generate and test hypotheses, and develop timely, evidence-based interventions.

Importantly, this study’s methodology and data pipeline can easily be replicated in other critical areas, including other health conditions, political or social science issues, or climate change. By leveraging this methodology, stakeholders engaged in these fields can gain a perspective on research trends, assess the current state of evidence, and anticipate future developments, thereby enhancing the efficacy of research and intervention strategies. Furthermore, future work in this area can extend the presented data in the pipeline with knowledge graphs and large language models (LLM). Such combinations can provide more accurate results. Knowledge graphs can establish complex relationships between concepts and entities in a literature corpus, providing a broader context for research results. Integrated LLM can enhance the capabilities of the classification process by capturing semantic nuances and complex patterns in text data. This advanced analysis can reveal hidden insights and trends in greater depth, enriching the understanding of the literature corpus.

Currently, a web application that uses natural language processing has been used to semi-automate systematic reviews and meta-analyses to reduce the time spent retrieving relevant articles to synthesise the results of research on one topic at one point in time (Chai *et al*., 2022^33^). However, researchers still need considerable time to screen abstracts to train the model (De Silva *et al*., 2024^34^). Using semantic modelling approaches our application can streamline and further enhance the review and evaluation of extant literature. Integrating our pipeline and dataset into an accessible interface could provide text summarisation with near real-time updates, marking a paradigm shift in the consolidation and dissemination of scientific knowledge. The platform could be developed with an evidence-quality assessment module to provide a user-friendly, evidence-informed question-and-answer query interface. By providing up-to-date, evidence-based insights, this tool empowers researchers and workers (e.g., healthcare professionals) to make informed decisions, ensuring that their practices reflect the latest and most effective interventions. This is particularly crucial in this current era in health care, where medical knowledge is expanding at an unprecedented rate, and staying abreast of the latest developments is both a challenge and a necessity for practitioners. However, it must be noted that this data management pipeline does not evaluate the quality of the publications, such as the use of the GRADE system^35^. Future iterations of the pipeline may include grading the quality of evidence and the synthesis of results to enable all stakeholders to interpret the findings.

From an educational standpoint, the dataset has the potential to revolutionise how evidence-based practices are taught and learned. Research-informed teaching ensures that the curricula and learning have currency in a rapidly changing world. These processes support educators to develop excellence in innovative teaching pedagogies and practices that positively impact the student experience. By exposing students to the dynamic nature of scientific evidence and highlighting historical and emerging trends, we can cultivate a receptive mindset to change and innovation. This approach aligns with Rogers’ Diffusion of Innovation theory^36^, suggesting that our system could catalyse the adoption of new ideas and behaviors among upcoming generations of professionals. By understanding that scientific knowledge is not static but evolves, we can prepare students to be adaptable, lifelong learners equipped to contribute to advancing their respective fields.

In conclusion, the development of this literature dataset, guided by the FAIR principles of findability, accessibility, interoperability, and reuse, represents a significant step toward improving open access to research related to COVID-19. By thematically classifying this extensive repository, we aim to provide researchers, policymakers, and funding bodies with critical insights into contemporary evidence-based solutions. The dataset empowers these stakeholders to stay informed about the latest interventions and effectively directs resources and governance to support and regulate these initiatives for the benefit of the community.

## Data Availability

The codes used in this publication for the data processing and the technical validation are available in Jupyter notebooks and accessible via the GitHub repository https://github.com/researchgraph/covid19-graph.
